# Comparative genome analysis of three classical *E. coli* cloning strains designed for blue/white selection: JM83, JM109 and XL1‐Blue

**DOI:** 10.1002/2211-5463.13812

**Published:** 2024-05-10

**Authors:** Stefan Achatz, Arne Skerra

**Affiliations:** ^1^ Lehrstuhl für Biologische Chemie Technische Universität München Freising Germany

**Keywords:** bacterial strain, *Escherichia coli*, F′‐plasmid, K‐12 restriction system, β‐galactosidase

## Abstract

The development of the *Escherichia coli* K‐12 laboratory strains JM83, JM109 and XL1‐Blue was instrumental in early gene technology. We report the comprehensive genome sequence analysis of JM83 and XL1‐Blue using Illumina and Oxford Nanopore technologies and a comparison with both the wild‐type sequence (MG1655) and the genome of JM109 deposited at GenBank. Our investigation provides insight into the way how the genomic background that allows blue/white colony selection—by complementing a functionally inactive ω‐fragment of β‐galactosidase (LacZ) with its α‐peptide encoded on the cloning vector—has been implemented independently in these three strains using classical bacterial genetics. In fact, their comparative analysis reveals recurrent motifs: (i) inactivation of the native enzyme via large deletions of chromosomal regions encompassing the *lac* locus, or a chemically induced frameshift deletion at the beginning of the *lacZ* cistron, and (ii) utilization of a defective prophage (ϕ80), or an F′‐plasmid, to provide the *lacZ∆M15* allele encoding its ω‐fragment. While the genetic manipulations of the *E. coli* strains involved repeated use of mobile genetic elements as well as harsh chemical or physical mutagenesis, the individual modified traits appear remarkably stable as they can be found even in distantly related laboratory strains, beyond those investigated here. Our detailed characterization at the genome sequence level not only offers clues about the mechanisms of classical gene transduction and transposition but should also guide the future fine‐tuning of *E. coli* strains for gene cloning and protein expression, including phage display techniques, utilizing advanced tools for site‐specific genome engineering.

## Graphical abstract

Based on their comparative genome analysis, JM83, JM109 and XL1‐Blue can be traced back to the classical research on the *lac* operon in the laboratory of F. Jacob. The Graphical Abstract depicts the genome map of JM83 superimposed on its colonies grown on an X‐gal agar plate transformed with a mixture of pUC19 plasmids with or without inserted heterologous DNA.

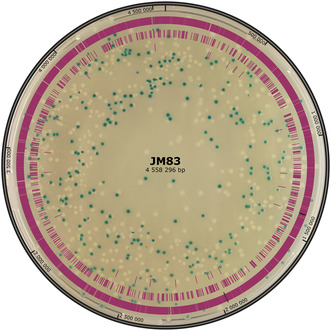

**Abbreviations**
aa: amino acid(s)
bp: base pair(s)
Hfr: high frequency of recombination
IS: insertion sequence
nt: nucleotide(s)

The selection and systematic optimization of bacterial laboratory strains for the cloning of heterologous DNA was instrumental in particular during the first decade of genetic engineering starting around 1980, after having established *Escherichia coli* K‐12 as a standard organism for genetic research [1]. Some of the developed strains, such as JM109 and XL1‐Blue [2, 3], serve as work horses for molecular biology even today. In this founding era of DNA cloning the efficiency of vector/insert ligation and bacterial transformation was a crucial aspect, and in this context the so‐called blue/white colony selection technique became popular and proved highly effective [4, 5].

The method of blue/white selection relies on the property of β‐galactosidase, the product of the *lacZ* gene, to hydrolyze the chromogenic substrate 5‐bromo‐4‐chloro‐3‐indolyl‐β‐d‐galactopyranoside (X‐gal) which results, after air oxidation, in an intensely blue and insoluble indigo dye. LacZ is a large homo‐tetrameric enzyme (EC3.2.1.23; 1023 amino acid [aa] residues per subunit—after processing of the initiator‐Met residue) with the peculiar feature that its N‐terminally truncated version, the so‐called ω‐fragment, has no catalytic activity, even though adopting a close to native quaternary structure [6]. However, non‐covalent complex formation with a rather short N‐terminal fragment—the α‐donor peptide, comprising residues ~13–50—restores a functional enzyme in a process called α‐complementation [7]. This phenomenon can be exploited for gene cloning purposes if the *E. coli* host strain expresses an N‐terminally truncated *lacZ* gene, especially the *lacZ∆M15* deletion mutant originating from the *E. coli* strain M15 [8], while the cloning vector carries a gene fragment comprising the *lac* ^p/o^ with the N‐terminal portion of the *lacZ* structural gene [9]. If, after transformation, colonies are grown on a culture plate containing X‐gal under conditions of recombinant gene induction they turn blue. On the other hand, if the N‐terminal *lacZ* gene fragment on the vector furthermore harbors a cloning site within the first codons, insertion of a heterologous DNA leads to a shift or interruption in the reading frame and, consequently, loss of a translated α‐peptide. As result, colonies carrying a plasmid with a successfully cloned DNA insert can be identified by their white color even in the presence of a majority of blue colonies that have just been transformed with the religated vector (see Graphical Abstract).

In the quest for host strains that facilitate such gene cloning experiments the following aspects were considered [2, 3]: (a) high transformation efficiency, (b) high plasmid or phage DNA yield, (c) reduced ability for DNA recombination (e.g. due to an inactivated RecA protein), (d) susceptibility to infection by filamentous phages and (e) blue/white colony selection to facilitate the isolation of recombinant plasmids or phage vectors. The male‐specific *E. coli* bacteriophage M13, a member of the Ff family of persistent filamentous bacteriophage (also including fd and f1) which possess a circular single‐stranded DNA genome, requires F‐pili (encoded on an F‐plasmid) for the infection of host cells [10]. In this context, the development of M13‐based cloning vectors [2, 9, 11] not only simplified single‐stranded DNA preparation for sequencing using the Sanger chain termination method [12, 13] as well as site‐directed mutagenesis via synthetic oligodeoxynucleotide hybridization and DNA mismatch repair [14, 15, 16]. Furthermore, these kinds of vectors subsequently provided the basis for the development of filamentous phage display techniques to select antibody fragments or other binding proteins from genetic libraries [17, 18, 19]. To this end, male *E. coli* strains such as JM109 and XL1‐Blue are still in wide use. Finally, (f) the absence of both generic and strain‐specific (K‐12) nucleases was considered helpful in order to prevent the ‘restriction’ of cloned heterologous DNA during bacterial transformation.

Aiming at such beneficial features for recombinant DNA cloning, sequencing, and mutagenesis, the strains JM83, JM109 and XL1‐Blue were constructed starting from more or less ‘natural’ *E. coli* K‐12 isolates—in part obtained after pretreatment with chemical or physical (X‐ray or UV) mutagens [20]—by using classical techniques of applied bacterial genetics [21]. So far, an analysis of their detailed genetic characteristics at the DNA sequence level has been missing but would be of high interest both for the continued application of these strains in molecular biology and for the future design of *E. coli* derivatives with advanced properties [22]. First, however, it is worth recapitulating the genesis of these laboratory strains, which were developed using procedures such as strain crossing, mating, conjugation, transposon mutagenesis, phage transduction as well as chemical mutagenesis.

JM83 (Table 1), in fact the first *E. coli* strain that was specifically developed for blue/white colony selection, was presented together with a series of other strains, including JM109, in the early 1980s [2, 9]. These newly constructed laboratory strains carried dedicated sets of mutations in particular to facilitate cloning of unmodified (non‐methylated) DNA and of repetitive sequences [2] and to allow blue/white selection in connection with the bacteriophage M13‐based as well as the high‐copy‐number pUC plasmid cloning vectors that likewise had been developed in the Messing laboratory [9]. While the latter report is often quoted as reference for JM83, the construction of this strain was actually described several years before in a less accessible publication [23]. According to this brief account, JM83 was generated on the basis of the strain CSH51 (*ara*, ∆(*lac*‐*pro*), *strA*, *thi*, ϕ80d*lac* ^+^) from the collection of the Cold Spring Harbor Laboratory (NY, USA), using a technique called homogenotization [21]. This procedure was employed to achieve the recombination of the *lacI* ^*q*^ *Z∆M15* gene variant present on the F′‐plasmid of the *E. coli* strain (BMH)71‐18 (∆(*lac*‐*pro*), *supE*, *thi* [F′ *proAB lacI* ^*q*^ *Z∆M15*]) [4] with the native *lacZ* gene present as part of the chromosomally integrated defective prophage ϕ80d, whereas the original *lac* operon had been deleted from the chromosome. Curing of the strain from the episome using acridine orange [21] resulted in the F^−^ genotype of JM83 (cf. Table 1). Of note, defective versions of the lysogenic bacteriophage ϕ80 were often used in classical bacterial genetics as transfer vectors for desired alleles between strains, especially in connection with the *lac* genes [24, 25, 26]. In fact, the progenitor strain CSH51, also referred to as X7700 [21], was described elsewhere [27] as an *ara* ^−^ and *str* ^*R*^ derivative of the classical LacZ‐deficient ∆(*lac*‐*pro*) strain X111 [28] which had been transduced with the defective recombinant phage ϕ80d*lac* [25].

**Table 1:** Genetic markers of *E. coli* strains investigated in this study.

K‐12 strain	Genotype	References
JM83	*ara*, ∆(*lac*‐*proAB*), *rpsL*(*str* ^R^), ϕ80d*lacZ∆M15*	[2]
‐''‐	*araC*, (∆*lac*‐*pro*)*X111*, *icd* ^+^, ϕ80d*lacZ∆M15*, *opgD*, *rfbD*, *gatC* ^+^, *gatY*, *yeiS*, *mglA*, *lrhA*, *evgS*, *xanQ*, *ttdB*, *yhdJ*, *rpsL*, *glpR* ^+^, *xylA*, *mtlA*, *rph* ^+^, *cytR*, *ytfI*, λ^−^	Revised, this study
JM109	*recA1*, *endA1*, *gyrA96*, *thi*, *hsdR17*, *supE44*, *relA1*, ∆(*lac*‐*proAB*), λ^−^ [F′, *traD36*, *proAB*. *lacI* ^*q*^ *Z∆M15*]	[2]
‐''‐	*sokC*, *sgrR*, *guaC*, ∆(*lfhA*‐*lac*‐*pro*‐*mhpF*), *supE*, *icd* ^+^, *narG*, *rssB*, *pspF*, *abgB*, *paaA*, *yncI*, *ydgI*, *rfbD*, *gatC* ^+^, *gatB*, *gyrA96*(*nal* ^*R*^), *menD*, *recA(L78P)*, *srlD*, *ygeH*, *endA1*, *ftsP*, *ttdB*, *yhcG*, *glpR* ^+^, *rph* ^+^, *yicL*, *bglH*, *rbsR*, *thiE*, *cadB*, *cpdB*, *fimE*, *hsdR17* (*r*_*k*_^−^, *m*_*k*_^+^), λ^−^ [F′::(*yafJ*‐*proBA*‐*lacI* ^*q*^ *Z∆M15*‐*yaiL*)]	Revised, this study
XL1‐Blue	*recA1*, *endA1*, *gyrA96*, *thi*, *hsdR17* (*r*_*k*_^−^, *m*_*k*_^+^), *supE44*, *relA1*, *lac* ^−^, λ^−^ [F′, *proAB*. *lacI* ^*q*^ *Z∆M15*, Tn*10*(*tet* ^*R*^)]	[3]
‐''‐	*sgrR*, *guaC*, *crl* ^+^, *lac* ^−^, *supE*, *pgaA*, *narG*, *rssB*, *tonB*, *abgB*, *paaA*, *yncI*, *kdgR*, *rfbD*, *gatC* ^+^, *gatB*, *gyrA96*(*nal* ^*R*^), *luxS*, *recA1*, *rpoS*, *ygeY*, *endA1*, *ftsP*, *ttdB*, *glpR* ^+^, *rph* ^+^, *bglH*, *rbsR*, *thiE*, *cadB*, *cpdB*, *fimE*, *hsdR17* (*r*_*k*_^−^, *m*_*k*_^+^), λ^−^ [F′::(*yafJ*‐*proBA*‐*lacI* ^*q*^ *Z∆M15*‐*yaiL*), Tn*10*(*tet* ^*R*^)]	Revised, this study

In contrast to JM83, the construction of JM109 [2] started from the *E. coli* K‐12 strain DH1 (*recA1*, *endA1*, *gyrA96*, *thi*, *hsdR17*, *supE44*, *relA1*, F^−^, λ^−^) previously developed by D. Hanahan [29, 30, 31], which was converted into DH1‐Tn*10*(*tet* ^*R*^)‐*recA* ^+^ via phage P1 transduction [21] using P1Cm1*clr*‐100 propagated in the strain TD1, a *recA56*, *srlC300*::Tn*10* derivative of MC4100. Subsequently, the ∆(*lac*‐*proAB*) chromosomal deletion was introduced by mating with the high frequency of recombination (Hfr) strain SL10 (Hfr H, *thi*, *sup*°, ∆(*lac*‐*proAB*), *galE*, ∆(*pgl*‐*bio*)), whose origin remains obscure. Curing from the Tn*10* transposon resulted in the strain JM106. The *recA* ^−^ phenotype was introduced into JM106 by transduction from the strain JC10240 (*recA* ^−^, *srlC*::Tn*10*), again followed by curing from the transposon, resulting in JM108. Finally, JM109 (Table 1) was obtained by mating JM108 with the earlier described male strain JM101 [23], leading to the acquisition of its episome. Together with the *lacZ∆M15* deletion, which enables blue/white colony selection via α‐complementation as described above, this mutated F′‐plasmid originally derived from (BMH)71‐18 carried the *traD36* mutation, which strongly reduces conjugation efficiency while still allowing infection by filamentous bacteriophage (such as M13). Besides, the presence of the *proBA* operon on the F′‐plasmid in conjunction with the chromosomal ∆(*lac*‐*proAB*) deletion permits selection on the episome via cultivation in a glucose minimal medium. Furthermore, JM109 constitutes a so‐called restrictionless host strain (*hsd*R17 = r^−^, m^+^), which means that any unmodified DNA cloned and propagated in this strain gets methylated but not degraded by the K‐12 type I restriction endonuclease, as will be described further below. In addition, the *rec*A mutation of JM109 diminishes recombination, which promotes the stability of both plasmid vectors and the cloned DNA.

While JM83 and JM109 were developed by Messing and coworkers in an academic setting, another laboratory strain allowing blue/white colony selection, XL1‐Blue [3], was developed at the company Stratagene Cloning Systems (San Diego, CA, USA), today part of Agilent Technologies (Santa Clara, CA, USA). This *E. coli* strain was constructed from an uncharacterized high transformation efficiency subclone of DH1 mentioned above. In the first step, a derivative dubbed BB1, carrying an inactivated *lacZ* gene, was selected via chemical mutagenesis using the acridine mutagen ICR‐191‐D, which causes frameshift mutations [21]. BB1 was initially conjugated with NM522 (∆(*lac*‐*proAB*), *thi*, *hsd∆5*, *supE* [F′, *proAB*. *lacI* ^*q*^ *Z∆M15*]), a *hsd* ^−^ derivative of (BMH)71‐18 [32]—whose F′‐plasmid carrying the *lacI* ^*q*^ *Z∆M15* allele also served for the construction of JM83 described above—and then functionally tested for α‐complementation using an M13 phage derivative encoding the α‐peptide. However, to facilitate selection on the presence of the F′‐episome—compared with propagation on minimal medium as previously practiced for JM109—a tetracycline resistance marker as part of the transposon Tn*10* was introduced by going one step back and mating NM522 with the Tn*10*‐carrying strain SV101 (*araD139*, ∆(*argF*‐*lac*)U169, *rpsL150*, *relA1*, *flbB5301*, *deoC1*, *ptsF25*, *rbsR*, *malPQ*::Tn*10*(*tet* ^*R*^)) [33]. Resulting SV101 cells harboring the F′‐episome from NM522 were selected on susceptibility towards infection by fd phage in a plaque‐forming assay. Subsequent transposition of the chromosomal Tn*10* into the episome was induced by repeated growth to stationary phase in liquid culture. The resulting modified F′‐plasmid carrying the *tet* ^*R*^ gene was isolated by conjugation with CSH10 (*trp*, *lacZ*, *strA*, *thi*) [21] which had been transformed with a plasmid carrying a kanamycin resistance gene. After that, CSH10 [F′, *proAB*, *lacI* ^*q*^ *Z∆M15*, Tn*10*] was conjugated with the strain BB1 from above to transfer the tetracycline‐selectable F′‐plasmid, finally yielding XL1‐Blue (Table 1).

Using state‐of‐the‐art bacterial genome sequence analysis, we here describe and compare the genetic changes at nucleotide (nt) level that have occurred in these three *E. coli* strains both for their chromosomes and their episomes, thereby revealing a series of previously unknown genotypic features.

## Materials and methods

The *E. coli* K‐12 strain XL1‐Blue was purchased as competent cells from Agilent Technologies (Lot# 6408908) whereas JM83 had been procured in 1986 from the strain collection of the Gene Center of the Ludwig‐Maximilians‐Universität Munich, Martinsried, Germany. DNA sequencing was performed on a NextSeq 500 instrument (Illumina, San Diego, CA, USA) with chromosomal DNA extracted using the phenol:chloroform:isoamyl alcohol method as well as on a PromethION instrument (Oxford Nanopore Technologies, Oxford, UK) with chromosomal DNA prepared using the Genomic‐tip 500/G kit (Qiagen, Hilden, Germany). The Illumina analysis resulted in 6 534 641 paired end reads for JM83, 1973 Mb in total, and 7 238 313 paired end reads for XL1‐Blue, 2185 Mb in total. The Nanopore analysis led to 68 519 sequence reads (up to 169 185 nt in length) for JM83, 906 Mb in total, and 207 689 sequence reads (up to 197 345 nt in length) for XL1‐Blue, 2771 Mb in total.

In the case of Illumina sequencing, the paired end reads (2 × 150 nt) were assembled to contigs using unicycler v0.4.8.0 software [34] whereas for Nanopore sequencing the raw sequence reads were assembled in parallel with the necat and flye program packages [35, 36]. The resulting contiguous sequences were visually inspected, annotated, and manually edited using snapgene software (GSL Biotech, Boston, MA, USA). For some ambiguous regions the original Nanopore sequence reads were compared against the assembled sequence with geneious prime software (Biomatters, Boston, MA, USA). This was particularly relevant for those genes which were duplicated from the *E. coli* chromosome on the F′‐plasmid of XL1‐Blue in order to assign mutations unambiguously to one or the other.

During alignment and gene annotation, reference was made to the following genome or plasmid sequences: *Escherichia coli* K‐12 MG1655 (GenBank ID: U00096.2, annotations of September 24, 2018 or of September 23, 2020) [37, 38], *Escherichia coli* K‐12 DH10B (https://asap.ahabs.wisc.edu) [26], *Escherichia coli* K‐12 plasmid F (GenBank ID: NC_002483.1) [39], MG1655 K12 plasmid F‐Tn10 (GenBank ID: MK492260.1) [40] and the *E. coli* bacteriophage ϕ80 (GenBank ID: JX871397.1) [41]. Gene annotations were adopted from these template sequences as appropriate. The assembled but not annotated chromosome and episome sequences of *E. coli* K‐12 JM109 were derived from GenBank [42] under the accession codes CP117962.1 and CP117961.1, respectively [43]. Of note, these latter sequences appear to be non‐curated and not fully devoid of bioinformatical artifacts as they commonly occur during initial assembly of raw sequence reads, such as missing nt in homo‐nucleotide repeat regions as well as a suspiciously large number of mutations in the ribosomal RNA genes (present in 7–8 copies on the bacterial chromosome). In the absence of available raw data, this prevented a more detailed analysis than presented here.

Raw sequence data as well as chromosome/episome assemblies for the *E. coli* K‐12 strains JM83 and XL1‐Blue elucidated in this study have been deposited at the National Center for Biotechnology Information (Bethesda, MD, USA) under NCBI BioProject accession number PRJNA740136 (JM83 chromosome: CP077969.2; XL1‐Blue chromosome: CP132550.1; XL1‐Blue plasmid F′: CP132549.1). The corresponding assembled and annotated genomic DNA sequences are also available in snapgene file format as Data [Link], [Link].

## Results and Discussion

### The JM83 genome

JM83 harbors a single circular chromosome of 4 558 296 bp which is largely homologous to the one of the *E. coli* K‐12 reference strain MG1655 [37, 38]. Apart from the larger gaps and insertions described in the following, there are in total 123 single nt substitutions (individually or as doublets) and 10 frameshift mutations in reading frames. One presumable triplet mutation in the *rrlD* gene for the 23S ribosomal RNA (nt 356–358: ATC→CAT) identically occurs in the genome sequence of XL1‐Blue described further below and may point to the independent derivation of these strains from *E. coli* K‐12 wild type that formed the basis of the Paris *lac* ^−^ strains (see below) compared to MG1655 [20, 38]. Some phylogenetic distance between JM83 and MG1655 is also evident from the significantly deviating number of insertion sequence (IS) elements (Table 2), in particular regarding IS*1* and IS*2*. Beyond that, the genome of JM83 reveals two dominant changes: (a) a large deletion covering the *lac* and *pro* operons and (b) insertion of a ϕ80d defective prophage.

**Table 2:** IS elements in the *E. coli* strains MG1655, JM83, XL1‐Blue and JM109.

IS element	MG1655	JM83	XL1‐Blue	JM109
IS*1*	8	5	8/2^a^	6/2^a^
IS*2*	7	9	7	7
IS*3*	5	4	5/3^a^	3/3^a^
IS*4*	1	2	1	1
IS*5*	12	11	16/1^a^	14/1^a^
IS*10*	–	–	2/3^a^	3
IS*30*	4	2	4/2^a^	2/2^a^
IS*150*	1	1	1	2
IS*186*	3	4	3	4
IS*600*	1	1	1	1
IS*609*	1	1	1	1
IS*911*	2	1	2/1^a^	1/1^a^
IS*X*	1	–	1/1^a^	–/1^a^
IS*Z*	1	1	1	1

^a^ IS element on episome (including the two IS*10* elements as part of Tn*10*).

First—as a necessary prerequisite for blue/white colony selection—the natural *lac* operon with the *lacZ* gene is absent in JM83, together with the *pro* operon, due to a large deletion (∆*lac*‐*proAB*) of 115 274 bp spanning the genes from *gpt* to *mhpC* (nt 256 104–371 377 in the MG1655 reference genome). This deletion starts almost directly upstream of the Lac repressor gene, *lacI*, and includes the entire cryptic prophage CP4‐6, several IS elements as well as the gene for the outer membrane porin PhoE. Apparently, this chromosomal deletion originates from the old‐established *E. coli* K‐12 derivative X111 (∆*proB*‐*lac*) [28], member of the ‘X’ series of heavily mutated *E. coli* strains [20] that were generated in the laboratory of F. Jacob in the early days of genetic research on the *lac* operon and its regulation. Of note, the alternative ∆*lacX74* chromosomal deletion described for the genome sequence of the remotely related laboratory strain DH10B (*araD139*, *∆(ara‐leu)7697*, *∆lacX74*, *galE15*, *galK16*, *galU*, *hsdR2*, *relA1*, *rpsL150(str* ^*R*^ *)*, *spoT1*, *deoR*, ϕ80d*lacZ∆M15*, *endA1*, *nupG*, *recA1*, *e14* ^−^, *mcrA*, *∆(mrr‐hsdRMS‐mcrBC*), λ^−^, F^−^) [26, 44], which also allows blue/white colony selection, originates from an independently isolated *lac*‐deficient *E. coli* K‐12 derivative, X74, from the same laboratory [25, 28].

**Fig. 1:**
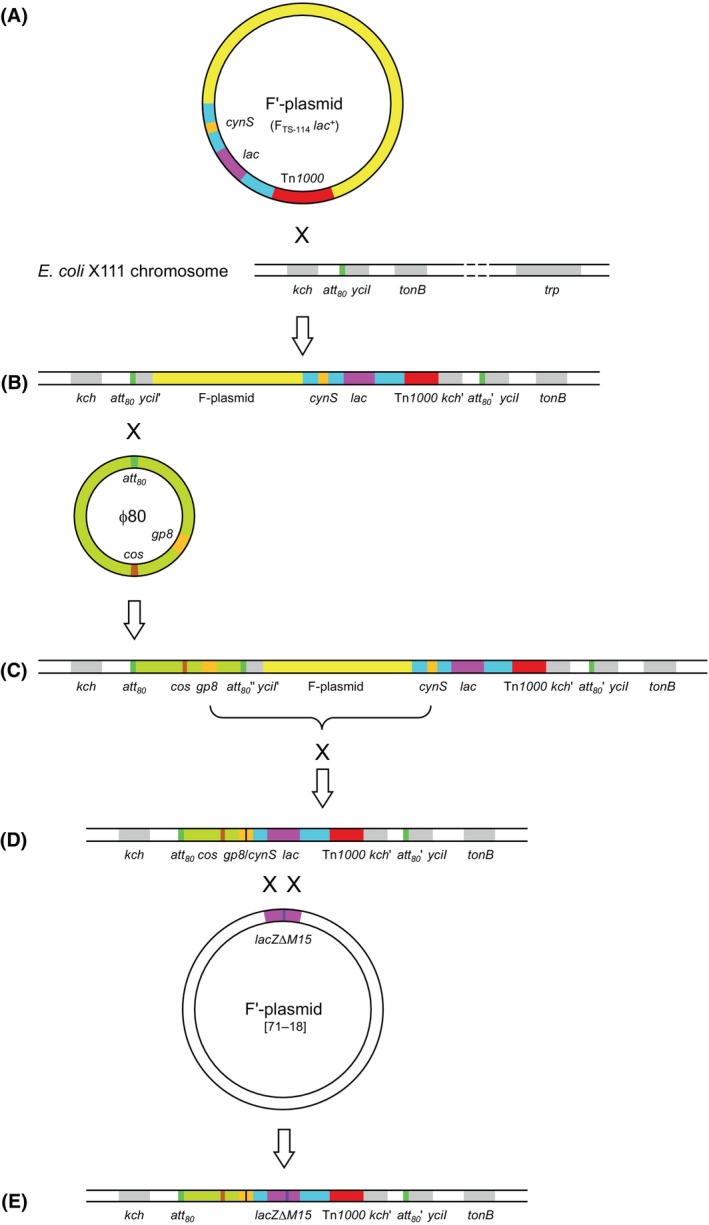
Potential mechanism how the defective prophage ϕ80d carrying the *lacZ∆M15* allele may have formed during construction of the *E. coli* strain JM83 based on its present genome sequence analysis (for details, see text). (A) Insertion of the F_TS‐114_ *lac* ^+^ episome via Tn*1000*‐mediated unspecific recombination (denoted by ‘X’) into the *kch* gene of the *lac*‐deficient X111 strain, which is accompanied by gene duplication of part of the *kch* gene as well as the *att*_*80*_ site and the *yciI* gene downstream. (B) Integration of the temperate bacteriophage ϕ80 into the first *att*_*80*_ site via homologous recombination. (C) Deletion between identical sequence stretches in the phage *gp8* gene and the *cynS* gene, upstream of the *lac* operon as part of the chromosomal insertion in the former F′‐plasmid, by homologous recombination. (D) Homogenotization, via double crossover recombination, between the F′‐plasmid 71‐18 and the ϕ80d*lac* region results in the chromosomal ϕ80d*lacZ∆M15* defective prophage in JM83 that encodes the LacZ ω‐fragment (E). Note that this scheme also explains the formation of the ϕ80*lac* transducing particles that are encoded between the pair of *att*_*80*_ sites (see the chromosomal segment in D) in its predecessor strains (e.g., CSH51 *alias* X7700), which get excised/replicated (and circularized) upon superinfection with native ϕ80 phage. As not all steps of the genetic manipulations have been accurately described in the literature it is possible that the order of some events may have differed in reality.

Second, there is a large insert, at the *att*_*80*_ site between the genes *kch* and *yciI* (Fig. 1), of the defective prophage ϕ80d [24, 41], which is identical to a similar truncated ϕ80 insertion that was found in DH10B [26]. The defective prophage comprises a 28 648 bp (62%) genome portion of the temperate lambdoid prophage ϕ80 [41]. This fragment is linked, via a 16 bp sequence overlap around the codon for Met320 in the ϕ80 major capsid protein, to a 12 762 bp piece of the *E. coli* chromosome (*cynS* to *mhpD*) that includes the *lac* region (in fact, the *lacZ∆M15* allele, see below). This portion is directly followed by the transposon Tn*1000* (5981 bp), which is also called the γδ sequence of the *E. coli* F‐plasmid. This mobile genetic element is known to function as attachment site for *recA*‐independent recombination between the episome and chromosome and, thus, can induce the formation of Hfr strains [45, 46]. Tn*1000* is followed by a 1006 bp truncated duplicate of the *kch* gene up to an almost exact second copy of the *att*_*80*_ site at the downstream end of *yciI*, which finally leads to the *tonB* gene further downstream on the chromosome (also known as phage T1 receptor) and, at some distance, to the *trp* operon. The exact match of the entire insert between the pair of *att*_80_ sites including the ϕ80d*lacZ∆M15* sequence that was previously elucidated for DH10B [26] can be explained by the circumstance that the latter had inherited it by way of recombination from the *E. coli* strain TB1, which is a *r*_*k*_^−^, *m*_*k*_^+^ derivative of JM83 [47].

Looking back into the history of *E. coli* strain construction, ϕ80*lac* transducing particles were initially obtained by Beckwith & Signer [25]. Based on earlier observations by Cuzin & Jacob [28], the initial step was the integration of F_TS‐114_ *lac* ^+^, a thermosensitive episome encompassing the *lac* operon [25, 48], into or close to the phage T1 receptor locus (*T1‐rec alias tonB*) near the natural ϕ80 attachment site, *att*_*80*_, on the ∆(*lac*‐*proAB*) strain X111 [28]. This resulted in several Hfr strains (among those, EC‐8), which were screened for *lac* transducing ϕ80 after infection and lysogenization with the bacteriophage (J. H. Miller, personal communication). According to Beckwith & Signer [25], the initially obtained transducing phage particles, ϕ80*lac*, carried a portion of the F‐episome in addition to the *lac* genes. In the light of the present genome sequence of JM83, this description suggests a series of recombination events as illustrated in Fig. 1:

(a) Chromosomal integration of the F′‐episome was mediated by recombination via the Tn*1000* transposon lacking sequence specificity [46], which occurred within the *kch* gene. This was accompanied by duplication of the adjacent part of this gene (at least 1023 bp, to the extent still preserved in JM83, see below), extending up to and probably beyond the *att*_*80*_ site at the downstream end of *yciI*. The resulting Hfr strain had the actual F‐plasmid sequence [10], followed by its own chromosomal insert including the *lac* operon (originating from F_TS‐114_ *lac* ^+^), oriented to the left of Tn*1000*. (b) Subsequent infection with wild‐type ϕ80 [24] led to lysogeny and insertion of the bacteriophage genome into the copy of *att*_*80*_ on the left side of the integrated episome. (c) Then, a homologous recombination event, apparently triggered by UV‐induced excision and multiplication of the bacteriophage [49], occurred between a 16 nt identical sequence stretch within the gene encoding the major capsid (head) protein of ϕ80 (*gp8*, similar to λ E, *N15*) [41] and within the *cynS* gene on the integrated F′‐plasmid, which is separated by just one gene (*cynX*) from the *lac* operon. During this step, more than one third of the ϕ80 genome (of in total 46 150 bp) [41] as well as the entire native F‐plasmid sequence and an unknown portion of its chromosomal insert were lost.

The result of this multiple genetic rearrangement corresponds surprisingly well to a published genetic map of this chromosomal region in the strain X7700 [50]. Besides, that paper also reports subsequent additional deletions extending further downstream to achieve transcriptional fusions between the *lacI* gene (directly upstream of *lacZ∆M15*) and the *trp* operon. Of note, while in the initial description of the *E. coli* X111 derivative EC‐8 the transposed *lac* operon from F_TS‐114_ *lac* ^+^ had been presented to the left of the *att*_*80*_ phage attachment site [25], a revised genetic map for the ϕ80*lac* lysogen [48] depicted it in line with the genomic sequence elucidated here. (d) The resulting defective prophage, including the *lac* ^+^ chromosomal fragment and the Tn*1000* transposon from F_TS‐114_ *lac* ^+^ as well as the *kdr* duplication up to the *att*_*80*_ copy downstream, was liberated as ϕ80*lac* transducing particle presumably via superinfection with the native ϕ80 phage [25], followed by a new transduction of the strain X111 (not shown in Fig. 1)—or a *str* ^*R*^ derivative thereof [27].

If it is true that ϕ80*lac* still carried part of the F′‐plasmid, which is difficult to assess from the original description of the genetic manipulations [25], steps (c) and (d) could as well have occurred in reverse order. Apart from that, the re‐transduction with ϕ80*lac*—while in principle not necessary, as all genetic elements were in place already after step (c)—and its integration into the single *att*_*80*_ site of an unaffected strain, such as X111, is supported by the circumstance that the flanking sequences on both sides of the two *att*_*80*_ copies present in JM83 are identical with the *E. coli* K‐12 reference genome. In fact, there is no compelling reason why the duplication of the flanking region (starting within the *kch* gene) induced by the initial chromosomal insertion of the F′‐episome via the Tn*1000* transposon in step (a) should have stopped precisely at the (non‐palindromic) *att*_*80*_ integration site for ϕ80.

**Fig. 2:**
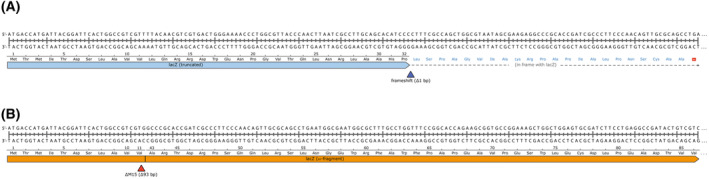
Comparison of the frameshift mutation in the non‐functional *lac*Z gene on the chromosome of XL1‐Blue (A) with the *lac*Z∆M15 deletion mutant, encoding the ω‐fragment of β‐galactosidase, on its episome (B). The deletion of a single C nt in the doublet of Pro codons (CCC‐CCT) at aa positions 32/33 of LacZ leads to a truncated reading frame of the chromosomally encoded enzyme (aa 1–32 plus 22 missense residues). On the other hand, the F′‐plasmid of XL1‐Blue carries an active gene for β‐galactosidase, however, with a 93 bp in‐frame deletion (known as ∆M15) which splices the codons for aa 11 and 43 in phase. This episomally encoded shortened version of the enzyme, the ω‐fragment, only becomes catalytically functional upon association with the so‐called α‐peptide (see text), which apparently needs to be longer than the N‐terminal 32 aa LacZ sequence that is still encoded on the chromosome.

Furthermore, the defective ϕ80d prophage carries the 93‐bp M15 deletion in its *lacZ* gene (Fig. 2), which splices codon 11 to codon 43 in phase, as previously described [51]. Hence, the *lacZ∆M15* allele present in its new position in the genome of JM83 encodes the ω‐fragment of β‐galactosidase, which enables the blue/white colony selection via α‐complementation. It is remarkable how precisely the *lacZ∆M15* deletion was transferred onto the chromosomally integrated ϕ80d*lac* prophage in a subsequent step (e) by means of homogenotization with the F′‐plasmid of the *E. coli* strain (BMH)71‐18 (as explained further above). Nevertheless, apparently the adjacent *lacI* ^*q*^ mutation [23] (see below) present upstream on the same episome was missed, in agreement with a later revised strain description of JM83 [9].

The resulting low number of Lac repressor molecules per cell expressed from the normal *lacI* gene (in its new location) explains the functioning of blue/white colony selection without supplementation of an inducer (such as isopropyl β‐d‐1‐thiogalactopyranoside, IPTG) to the culture plate when transforming JM83 with high‐copy‐number cloning plasmids like those from the pUC family [9]. In this setting, the many copies of the *lac* ^p/o^ effect almost constitutive expression of the *lacZ* α‐peptide from the plasmid. From a practical perspective, the conspicuous leakiness of the *lac* ^p/o^ on high‐copy‐number expression vectors can lead to a phenomenon of host cell toxicity in particular when dealing with secreted heterologous gene products. This had prompted us to clone a viable *lacI* gene directly on the expression plasmid and to apply glucose‐mediated catabolite gene repression when developing the first system for the biosynthesis of functional antibody fragments in *E. coli* [52, 53].

Beyond these two large chromosomal rearrangements, the genome sequence of JM83 reflects the genetic markers as described in the classical publication [2] (see Table 1). The reading frame for the DNA‐binding transcriptional dual regulator AraC reveals a nonsense mutation (opal stop codon at aa position 91). The 30S ribosomal subunit protein S12 encoded by the *rpsL* gene carries the selected spontaneous missense mutation K47N (AAA→AAC), which leads to resistance against the antibiotic streptomycin (*strA* or *str* ^*R*^). Interestingly, common resistance‐conferring *rpsL* mutations known so far from *E. coli* K‐12 isolates are K42T and K87R [54] whereas the cloning strain DH10B reveals the aa exchange K43R [26]; hence, JM83 exhibits an alternative *str* ^*R*^ antibiotic marker.

Finally, the *thiCEFGH* operon [55, 56] appears intact in the JM83 genome, however, with the exception of the mutation D70A (GAT→GCT) in *thiE* encoding the thiamine phosphate synthase. This non‐conservative aa exchange lies at the entrance to the active site of the enzyme [57] and may influence substrate binding. Therefore, it would be interesting to investigate via enzymatic analysis to which extent this mutation may affect the catalytic activity of ThiE. Of note, the initial annotation of thiamine auxotrophy [9, 23] was revised in subsequent genetic descriptions of JM83 [2, 56].

Further to these known genotypic traits of JM83, a number of mutations in other genes compared with the MG1655 reference genome that were not previously anticipated have become apparent from its genome sequence analysis (Table S1), even though no fundamental gene defects seem to be present. Together with a significant number of mostly silent point mutations, these deviations from the wild‐type genome sequence presumably reflect the history of harsh UV and X‐ray mutagenesis treatments in the pedigree of this bacterial laboratory strain [20].

Among these genetic aberrations, the *icd* gene for isocitrate dehydrogenase lacks the inserted cryptic prophage e14, one of the nine cryptic prophages described for the genome of the wild‐type *E. coli* K‐12 strain MG1655 [37, 58], thus encoding a fully functional gene product. In MG1655, insertion of e14 into the *icd* reading frame after codon 362 led to the duplication of a 165 bp segment (dubbed *icdC*) on the other side of the lysogen. This partial gene duplication is accompanied by 12 nt exchanges (resulting in two conservative aa replacements, both Glu to Asp) but an intact reading frame for the 54 C‐terminal residues of the Icd enzyme encoded upstream of e14 [59]. This pattern of mutations in the *icd* sequence of MG1655 becomes evident here by comparison with the uninterrupted gene of JM83—and also with the one of DH10B described before [26].

Furthermore, in JM83 the *rph* gene is devoid of the frameshift deletion known for MG1655 (harboring the *rph‐1* allele) and encodes an intact ribonuclease PH (UniProt ID: http://www.uniprot.org/uniprot/P0CG18), which plays an important role in tRNA 3′‐end maturation. In contrast, the truncated gene product in MG1655, as well as in its sibling W3110 [38], lacks RNase PH activity. This leads to pyrimidine starvation in these strains due to low levels of orotate phosphoribosyltransferase (PyrE), apparently since close coupling between transcription and translation is needed to support optimal levels of transcription past the intercistronic *pyrE* attenuator [60]. The intact *rph* reading frame in JM83 additionally confirms the independent derivation of the Paris *lac* ^−^ strains from *E. coli* K‐12 wild type [20].

### The XL1‐Blue genome

The chromosome of XL1‐Blue (4 633 417 bp) is largely homologous to the one of the *E. coli* K‐12 reference strain, MG1655, even though it exhibits a remarkable number of point mutations (in total 345 substitutions, as single or double nt exchanges) plus 8 frameshifts. Both likely result from the harsh chemical mutagenesis that was performed during its selection on the LacZ^−^ phenotype as mentioned above.

In fact, the chromosomal *lac* operon is almost fully present, and there is no extended deletion as in the case of JM83 and JM109 (see below); instead, there is a single C deletion in the doublet of Pro codons (CCC‐CCT) at aa positions 32/33 of LacZ (Fig. 2)—further accompanied by a point mutation in codon 333 downstream. The frameshift and resulting missense aa sequence starting at codon 33, followed by a first stop codon at position 55, renders the chromosomal *lac*Z coding region non‐functional. Obviously, the short correctly translated N‐terminal stretch of 32 aa is insufficient to yield a functional α‐peptide (146 residues normally), in line with recent insights from the X‐ray structural analysis of β‐galactosidase [6]. Importantly, this lethal frameshift mutation in the chromosomal *lac*Z gene forms the basis for the α‐complementation mediated by the LacZ ω‐fragment encoded on the F′‐plasmid, as will be described further below.

All other previously described genetic markers of XL1‐Blue (see Table 1) [3] can be explained by mutations at the genome sequence level. For example, the genotypic feature *supE44*—more generally designated *supE* [61]—was found in the duplicate copy directly downstream of the *glnV* tRNA gene, previously annotated *glnX*, an allele of tRNA_2_^Gln^ [62]. Transition of nt 35 from G to A leads to a change at the third position of the anticodon (CUG→CUA), thus encoding a suppressor tRNA which recognizes the amber stop codon (UAG). The partial suppression of this kind of stop codon (and its translation into Gln), introduced at the junction of gene fusions between a binding protein and the minor M13 phage coat protein pIII, has been employed for phagemid display experiments [17, 63] and, therefore, prompted the use of XL1‐Blue and related strains.

Otherwise, the *gyrA96* marker of XL1‐Blue is due to a single nt mutation at codon 87 (GAC→AAC) of the *gyrA* gene. The corresponding D87N aa exchange in the DNA gyrase confers resistance to nalidixic acid (*nal* ^*R*^), a potent gyrase inhibitor. While this mutation is commonly found in quinolone‐resistant clinical isolates of *E. coli* [64], the *gyrA96* mutation was initially selected as a spontaneous mutation conferring nalidixic acid resistance during construction of the cloning strain DH1 [29], the ancestor of XL1‐Blue (as explained further above).

Likewise, DH1 had been selected for the *recA1* as well as the *endA1*, *relA1* and *hsdR17* (*r*_*k*_^−^, *m*_*k*_^+^) genotypes [29]. Indeed, in the genome sequence of XL1‐Blue the *recA* gene carries a single nt exchange within codon 161 (GGC→GAC). The resulting aa substitution G161D leads to a loss of function for the DNA recombination/repair protein RecA, as also seen in the genome sequence of DH10B, another descendant of DH1 [26]. While this mutation improves clone stability by inhibiting the homologous recombination system, it also has the less desirable effect that concatenated plasmids do not resolve to monomers [29]—which can complicate site‐directed mutagenesis experiments by provoking the appearance of clones carrying mixed plasmid populations. Similarly, the *endA* gene carries a single nt substitution within codon 208 (GAG→AAG) that results in the aa exchange E208K, as also described for DH10B [26]. This missense mutation inactivates the periplasmic DNA‐specific endonuclease I, thereby enhancing DNA stability during transformation.

Finally, the *hsdR17* (*r*_*k*_^−^, *m*_*k*_^+^) genotype is evident from a stop codon at aa 363 in the *hsdR* gene which encodes the endonuclease component of the type I restriction/modification system EcoKI [65]. The other two genes of the *hsd* (‘host specificity for DNA’ or ‘host specificity determinant’) locus remain intact and active, i.e. *hsdS* encoding the subunit responsible for DNA sequence specificity and, in particular, *hsdM* encoding the subunit that has methyltransferase catalytic activity. While these three structural genes are arranged directly adjacent in the order *hsdR*, *hsdM*, and *hsdS* and all are transcribed in the downstream direction, they do not necessarily form one operon, as there is a second promoter downstream of *hsdR* which initiates transcription of *hsdM* and *hsdS* [66]. Otherwise, if part of the same operon, the nonsense mutation in *hsdR* would lead to a 'polar effect' and, thus, diminished (or even missing) expression of the two downstream cistrons.

Apart from classical cDNA cloning, the resulting *m*_*k*_^+^ phenotype has become useful with the advent of the polymerase chain reaction (PCR) for the amplification of genes or reverse‐transcribed RNA as well as for the assembly of synthetic genes [67, 68], as the resulting enzymatically generated DNA is unmethylated. Consequently, if the 5′‐AACN_6_GTGC‐3′ recognition motif [69] occurs, this unmodified DNA would be degraded (‘restricted’) by the EcoKI endonuclease holo‐enzyme directly after transformation. Since XL1‐Blue still synthesizes the necessary subunits for the corresponding methylase [65], including HsdS, any plasmid DNA isolated from it can subsequently be used for the efficient transformation of other *E. coli* K12 strains that carry the intact (*r*_*k*_^+^, *m*_*k*_^+^) restriction system.

Finally, at first glance the operon responsible for thiamine biosynthesis, *thiCEFGH* [56], appears intact. However, compared with the reference strain MG1655, there are three point mutations: a silent mutation (GGT→GGC) at codon Gly166 of *thiE*, a G129R missense mutation (GGA→AGA) in *thiE* and a E134K missense mutation (GAG→AAG) in *thiF*, the latter encoding the sulfur carrier protein ThiS adenylyltransferase. Importantly, the original small Gly residue at position 129 in the *thiE* cistron directly lines the active site of the thiamine phosphate synthase, ThiE [57], and its replacement by the large positively charged Arg side chain would lead to a severe clash with the bound substrates. Hence, it can be assumed that this mutation is more severe than the D70A aa exchange discussed in the context of JM83 and renders ThiE in XL1‐Blue enzymatically inactive.

Unexpectedly, the gene for the GDP/GTP pyro‐phosphokinase RelA shows an intact reading frame, without disruption by IS*2* as described for DH10B [26], and it does not reveal any nt exchange compared to the reference strain MG1655. In fact, in the description of the ancestor strain DH1 the *relA1* genetic marker was reported by D. Hanahan with a question mark [29], and its recently disclosed genome sequence [30, 31] reveals an uninterrupted reading frame identical to the one seen here for XL1‐Blue. Therefore, based on the present genome sequence analysis, the established genetic markers of XL1‐Blue need substantial revision (see Table 1), also in the light of the considerable number of additional genes that are affected by single nt substitutions, frameshift mutations or recombination of mobile genetic elements (see Table 2 and Table S2). Notably, indications for unanticipated genetic aberrations in XL1‐Blue became already apparent from a comparative proteome analysis of this *E. coli* laboratory strain [70].

While probably less relevant for the native set of *E. coli* genes, there is a large deletion within the sequence of the defective lambdoid prophage DLP12, downstream of *argU* [71]. This deletion ranges (in upstream direction) from codon 51 of the putative integrase, IntD, almost up to the gene for the putative phage lysis protein, EssD, and is accompanied by an insertion of IS*5*. For comparison, in the genome of MG1655 there is an insertion of IS*5* close to the C‐terminus of the putative outer membrane porin, NmpC, downstream of the *essD* gene, and another insertion of IS*3* in the gene for the prophage protein RenD. Hence, it seems that XL1‐Blue exhibits an advanced level of genetic degeneration of this defective prophage [58].

Likewise, the cryptic prophage e14 has two defects in comparison with the MG1655 genome [58]: an insertion of IS*3* into *intE*, the gene for its putative integrase, and a precise inversion of a 1797 bp segment ranging from within the *ycfK* gene to within the *stfE* gene, whereas the pair of *tfaE* and *tfaP* genes in between, oriented in mutually opposite directions and partially overlapping at their 3′‐ends, remains intact. In spite of the insertion of the modified e14 into the *icd* gene, XL1‐Blue still encodes a functional gene product (see the explanation above for JM83). Beyond these two genetically altered prophages, the cryptic prophage CPZ‐55, inserted between the *eutA* and *eutB* genes in MG1655 [58], is missing in XL1‐Blue.

As another class of mobile element, two identical copies (in the same orientation) of the insertion sequence IS*10R* (1329 bp) are found in the genome of XL1‐Blue: (a) insertion into the *tonB* gene at codon Ser222 and (b) insertion into *yncI* at codon Cys262 (of note, the reading frame for the putative transposase YncI is already truncated by a stop codon at aa position 249 both in the genome of MG1655 and in the one of JM83). This insertion sequence originates from the pair of almost identical flanking inverted repeats, IS*10*‐Left and IS*10*‐Right, on both sides of the transposon Tn*10*, a composite mobile genetic element [72, 73]. The actual mobile element of Tn*10* is IS*10R*, which carries the gene for the IS*10*‐Right transposase, thus also enabling its escape and migration as an independent entity [73]. In contrast, the IS*10*‐Left transposase has been reported to be inactive [74], apparently due to just a few deviating nt positions, and is not found in the genome of XL1‐Blue. While Tn*10* is not a common component of *E. coli* genomes [73], and IS*10* sequences are in fact absent both from the reference strain MG1655 and from JM83, two copies of IS*10R* and one of IS*10L* have been described for DH10B [26], whereas one copy of IS*10R* and two of IS*10L* can be identified in the genome of JM109 (see below). On the other hand, IS*10R* naturally occurs as part of Tn*10* on the XL1‐Blue F′‐plasmid, together with two copies of IS*10L*, as will be described in the following.

### The F′‐plasmid of XL1‐Blue

Compared with the sequence of the well known native F‐plasmid of *E. coli* K‐12 [10, 39, 75] this episome has more than double the size, with 242 042 versus 99 159 bp. The entire sequence of the natural F‐plasmid forms part of it and is almost exactly conserved – with just about 10 nt mutations (mainly substitutions as well as a few insertions/deletions in non‐coding regions). However, there is a large insertion flanked by two identical copies of the IS*3* mobile element that interrupts its *finO* gene (encoding an RNA binding/chaperone).

**Fig. 3:**
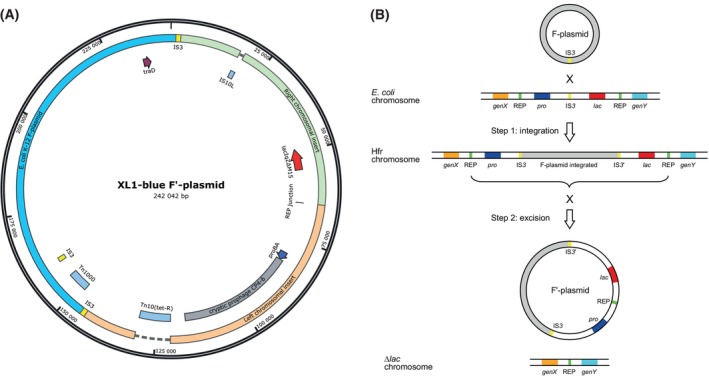
Map of the F′‐plasmid of XL1‐Blue and mechanism of its formation. (A) Two large pieces of chromosomal DNA from *E. coli* K‐12 (‘Right’ and ‘Left’) are inserted into one of the IS*3* elements of the original F‐plasmid, which became duplicated during this step. These two segments—initially contiguous on the *E. coli* chromosome, but in reverse order (see text)—are joined via a short homologous sequence (‘REP junction’). On top of that, there is an insertion of the transposon Tn*10* which was introduced later and confers tetracycline resistance (*tet* ^*R*^). The *lac* operon with the M15 deletion, thus encoding the LacZ ω‐fragment, is situated in the right chromosomal segment. The location of the *traD* gene, which is mutated in the F′‐plasmid of JM109, is labeled for comparison. (B) Generation of the F′‐plasmid from the natural episome of *E. coli* K‐12 via homologous recombination with its chromosome in two steps: (a) insertion via recombination with the IS*3* element in the vicinity of the *lac* locus, forming a Hfr strain; (b) excision mediated by recombination between two repetitive DNA segments in both flanking regions, each at some distance. In this way, the episome acquires surrounding chromosomal genes, here including the *lac* operon and also the *pro* operon. At the same time the entire corresponding region gets deleted from the chromosome. Interestingly, even very short repeated DNA segments (7–9 nt) seem to be sufficient to trigger this kind of episome excision, thus explaining the generation of a series of known *lac*‐deficient *E. coli* strains (see text).

This extended insertion is dominated by a single contiguous region from the MG1655 reference genome, yet in rearranged order, as well as the presence of the class I transposon Tn*10*, together with a second copy of IS*10L* (Fig. 3). The genomic insert, divided into two portions, covers almost exactly nt 244 143–377 401 of MG1655, beginning in a repeat region (REP*17a*) upstream of the *yafJ* gene and ending in a homologous repeat region (REP*32b*) downstream of *yaiL*. Interestingly, this piece of genomic DNA is larger than the overlapping chromosomal deletions observed in JM83 and in JM109 (see below). Apart from very few point mutations, only the IS*1* insertion sequence in the *crl* gene of MG1655 (but not XL1‐Blue) is missing on this F′‐plasmid. Roughly in the middle of this genomic region, there occurs an IS*3* insertion (corresponding to nt 315 229–316 483 in the MG1655 chromosome) at the junction between the *eaeH* and *ykgA* genes. This divides the chromosomal fragment into a left and a right portion, which are separately linked to the sequence of the original F‐plasmid (see Fig. 3). The left portion contains the *pro* operon—as well as the cryptic prophage CP4‐6—whereas the right portion harbors the entire *lac* operon including, importantly, the *lacZ∆M15* and *lacI* ^*q*^ mutations.

This peculiar arrangement of the two chromosomal DNA portions, flanked by copies of IS*3* on both sides, indicates a previous genomic insertion of the F‐plasmid mediated by one of its own (two) IS*3* mobile elements, followed by a type II excision encompassing flanking chromosomal genes. In other words, the native F‐plasmid presumably has integrated into the bacterial genome by homologous recombination via the IS*3* insertion sequence—instead of Tn*1000* as described in the context of JM83 further above—at the connection between the chromosomal *eaeH* and *ykgA* genes, thus forming a Hfr strain. Subsequently, the episome was excised while encompassing the chromosomal regions on both sides, whose termini became joined upon circularization. This latter homologous recombination step was apparently facilitated by the circumstance that the extreme ends of the left and right portions of the genomic DNA lie in two mutually highly similar repeat regions (REP*17a* and REP*32b*, respectively), which merely differ in 3 nt positions within an otherwise identical 34 bp stretch.

On the resulting F′‐plasmid, the left chromosomal insert is interrupted by the class I transposon Tn*10*, which was subsequently introduced by intent [3] in order to insert the tetracycline operon with the resistance gene *tetA* [72, 74]. Interestingly, a second copy of the IS*10*‐Left transposase is also seen within the right chromosomal insert region (cf. Fig. 3). This indicates that the transposition may have happened in two stages, with one copy of IS*10L* left behind, as this mobile genetic element by itself should be inactive—in contrast to its sibling IS*10R* as explained above. This order of events is supported by the fact that both Tn*10* and the isolated IS*10L* are missing on the otherwise almost identical F′‐plasmid of the strain JM109 (see below).

The ∆M15 in‐frame deletion (93 bp) in the *lacZ* gene, which forms the basis of α‐complementation, is identical to the one described above for the ϕ80d*lacZ∆M15* defective prophage as part of the JM83 genome and apparently originates from the same old *lac* deletion strain of *E. coli*, M15 [8]. However, here the promoter of the gene encoding the Lac repressor furthermore exhibits the ‘quantity’ mutation, *lacI* ^*q*^ [76]. This small additional genetic change explains why blue/white colony selection with XL1‐Blue requires the supplementation of an inducer of *lac* gene expression, such as IPTG, to the agar culture medium (together with the X‐gal substrate) since even on high‐copy‐number plasmids the *lac* ^p/o^ that controls the expression of the LacZ α‐peptide is fully repressed in this genetic background.

In summary, the DNA sequence of the XL1‐Blue F′‐plasmid is consistent with its origin from the F′‐plasmid of the *E. coli* strain (BMH)71‐18 (see above). Unfortunately, the genesis of (BMH)71‐18, first referred to in a publication by J. Messing [4], is not well documented. Initially dubbed 71‐18, its F′ *lac pro* episome, with the I^q^ mutation in the promoter of the *lacI* gene and the M15 deletion in *lacZ*, was constructed by J. Miller (personal communication) in a collaboration with B. Müller‐Hill, who subsequently included it in his strain collection with the designation BMH71‐18 [77]. With the acquisition of the Tn*10* transposon later on, the DNA sequence of the F′‐plasmid of XL1‐Blue fully conforms with its original genetic description [3] (see Table 1).

### Comparison of the chromosome and episome of JM109 with XL1‐Blue

Considering the history of strain construction, JM109 occupies an intermediate position between JM83 and XL1‐Blue. Consequently, a comparison with its recently elucidated genome sequence, both of the chromosome and the episome [43], appears of interest. Again, the sequence of the JM109 chromosome is highly similar to the *E. coli* K‐12 reference genome of MG1655 and, moreover, it resembles in several aspects those of the derivatives JM83 and XL1‐Blue. Similar to JM83, JM109 carries a 124 324 bp deletion of the entire *lac* operon region, here starting in the *lfhA* gene and ending at the junction (4 nt overlap) between *mhpF* and *mhpE* (nt 249 546–373 869 in the MG1655 genome). Of note, there is a short sequence repeat on both sides (GCGGCAT), which may have triggered a homologous recombination (as will be discussed further below). However, the chromosomal deletion in JM109 is larger, at both ends, than the one seen in JM83 which can be traced back to the *E. coli* strain X111 [28]. Interestingly, it also differs from the ∆*lacX74* chromosomal deletion [28] as it has been elucidated for DH10B [26] and from the ∆(*argF*‐*lacZYA*)*U169* deletion described for DH5α [78]. A BLAST search in GenBank with the 2 × 24 nt sequence covering the new DNA junction present in the chromosome of JM109 did not lead to any meaningful results. Hence, the origin of this alternative ∆(*lac*‐*proAB*) deletion, which was crossed in from the poorly described strain SL10 [2], remains in the dark. Possibly, it could stem from one of the old sibling ∆*lac*‐*pro* strains of X111, such as X103 or Y23 [28].

Apart from that, the genome sequence of JM109 shows close resemblance to the one of XL1‐Blue (Table S3). In particular, it carries exactly the same point mutations in those genes which make it attractive for gene cloning and phage display applications: *hsdR17*, *endA1*, *gyrA96* and *supE44*. In the latter context, the suppressor gene *supE* is crucial as many phagemid display vectors carry an amber stop codon in the gene III fusion protein with an antibody fragment—or alternative binding protein—whose partial translation (as Gln residue) leads to the parallel biosynthesis of the soluble binding protein together with its gene III fusion [17, 63], as explained already for XL1‐Blue above.

Similar to XL1‐Blue, the *relA* gene appears intact in JM109 whereas the *thi* operon exhibits the same inactivating G129R mutation for the ThiE enzyme, together with the E134K missense mutation in the *thiF* gene (but without the silent mutation within codon Gly166 of *thiE*). Apparently, all these genetic markers were inherited from DH1 during the construction of JM109 as explained further above. However, the *recA* ^−^ genotype was introduced independently [2] and, indeed, this gene carries the missense mutation L78P instead of G161D (as in XL1‐Blue or DH10B, see above). On the other hand, most of the many point mutations seen for XL1‐Blue, which were probably acquired when selecting for the *lacZ* frameshift on its chromosome via chemical mutagenesis, seem to be absent in the JM109 genome.

Finally, like XL1‐Blue, JM109 harbors an F′‐plasmid. The sequence of its episome is almost identical (see Fig. 3), with the exception of the Tn*10*(*tet* ^*R*^) and the IS*10L* insertions that were specifically introduced during the strain construction of XL1‐Blue. In addition, however, the F′‐plasmid of JM109 harbors the *traD36* mutation, which had been incorporated by crossing the allele from the mutated episome JCFL36 [79] with the F′‐plasmid of the strain (BMH)71‐18 (see above) during the construction of JM101 [23]. According to J. Messing, this mutation interferes with transfer of the F′‐plasmid, thus hindering horizontal gene transfer via conjugation—as recommended by the U.S. National Institutes of Health (NIH, Bethesda, MD, USA) in the early days of gene technology—but still allows infection by M13 and propagation of recombinant filamentous phage vectors.

Astonishingly, it seems that the *traD36* mutation has not been characterized to date in spite of the wide use of this F′‐plasmid for cloning purposes and phage display in combination with the strain JM109 or its relatives, including TG1 [80], and elucidation of the *traD* gene sequence itself many years ago [81]. From a sequence comparison with the F′‐plasmid of XL1‐Blue, and also with the wild‐type K12 F‐plasmid [75], it appears that there is just a single nt mutation (GAG→AAG) leading to the aa exchange E253K. This mutation is identically found in the recently disclosed episome sequence of TG1 [82]. The *traD* gene encodes the so‐called coupling protein, a hexameric ring ATPase that forms the cytoplasmic face of the pore during F‐plasmid‐mediated bacterial conjugation and tightly interacts with the relaxosome component TraM [83]. According to its structural homology with the hexameric conjugation protein TrwB from the R388 conjugative plasmid of *E. coli* [84] the mutated position (corresponding to E190 in TrwB) is located in the nucleotide‐binding domain of TraD. Thus, the charge reversal (negative to positive) associated with the *traD36* mutation probably compromises its function as ATP‐driven pump for DNA transfer but it should not affect the pilus formation itself [10].

Of note, the F‐pilus as encoded on the F‐plasmid can promote DNA transfer in two directions: first, export of its single‐stranded DNA copy from the cytoplasm during horizontal gene transfer to recipient cells and, second, import of DNA via attachment of filamentous phage from outside. In contrast to the ATP‐driven DNA export during bacterial conjugation, a filamentous phage just utilizes the pilus tip as primary attachment site, which is followed by pilus retraction [85, 86], whereas the pore‐forming and proton‐dependent *tol* system of the host cell then serves for the import of its circular single‐stranded DNA genome [87]. This explains why the DNA transfer system of conjugative plasmids, here affected by the *traD36* mutation, is unimportant for the infection process of filamentous phage such as M13—which constitutes a crucial step in bacterial phage display technology.

## Conclusions

The comparative genome sequence analysis of the three *E. coli* K‐12 laboratory strains JM83, JM109 and XL1‐Blue reveals individual histories of enforced physical/chemical mutagenesis and the use of mobile genetic elements—‘hopping genes’—to achieve essentially the same goals: first, beneficial properties for recombinant DNA cloning and propagation and, second, the possibility of blue/white colony selection following transformation with high‐copy‐number plasmids that encode a LacZ α‐peptide interrupted by a cloned gene. To this end, all three strains utilize, either on their chromosome or on an episome, genetic traits that can be traced back to the classical work on the elucidation of the structure and regulation of the *lac* operon in the laboratory of F. Jacob, further complemented by fundamental experiments in bacterial genetics conducted by J. Beckwith and others [21, 28, 48].

JM83 was the first strain in this series that was specifically constructed for cloning purposes by J. Messing [23]. It made use of the large ∆(*lac*‐*proAB*) deletion from the initial *E. coli* derivative X111 [28] while introducing the tiny *lacZ∆M15* deletion from the strain M15 [8]—thus encoding the LacZ ω‐fragment—by means of the integrated defective prophage ϕ80d*lac* [25]. Although JM83 itself is less widely used, its direct descendant TB1 [47] or its more remote progeny such as DH10B [44] commonly serve as cloning strains until today. Beyond purposes of recombinant DNA manipulation, JM83 has served as a bacterial host for efficient periplasmic protein secretion in our hands [88].

In comparison, XL1‐Blue still seems popular, probably due to a series of inactivated genes that facilitate the efficient and stable transformation with plasmid DNA and, furthermore, allow infection with filamentous phages: in particular, *hsdR17*, *endA1*, *recA1*, *gyrA96*, *supE44* as well as the F′‐plasmid [3]. In this case, blue/white colony selection is enabled by a copy of the *lacZ∆M15* allele on the episome, which itself was made selectable by introduction of the tetracycline resistance gene. In contrast to JM83, XL1‐Blue does not harbor a large ∆(*lac*‐*proAB*) deletion but has its chromosomal copy of the *lacZ* gene inactivated by a frameshift mutation at the beginning of the coding region (see Fig. 2). Introduction of this genetic trait by heavy chemical mutagenesis came at the expense of an impressive number of chromosomal mutations and genes affected (see Table S2), which likely explains the relatively slow growth of this laboratory strain and its inferior performance in recombinant protein production.

Finally, JM109, an independently developed strain from the laboratory of J. Messing [2]—generated after JM83 but prior to XL1‐Blue—reveals mixed genetic features. On the one hand, like JM83 it carries a large ∆(*lac*‐*proAB*) deletion, even though more extended, and on the other it encodes the LacZ ω‐fragment as a prerequisite for blue/white selection on its episome, similar to XL1‐Blue. Due to their common DH1 ancestry, JM109 shares most of the auxiliary features for DNA cloning with XL1‐Blue: such as lack of K‐12 restriction, of RecA‐mediated homologous recombination and of the periplasmic endonuclease. In contrast, its genetic background appears more healthy in the light of many missing point mutations, even though the large ∆(*lac*‐*proAB*) deletion is accompanied by a loss of several relevant genes. Fortunately, many of these, such as *proBA*, are complemented by the large chromosomal insertion in its F′‐plasmid. Importantly, this confers the ability of JM109 to grow on glucose minimal medium (if supplemented with thiamine) while providing selection on the presence of the episome at the same time.

Regarding the origin of the different large genomic deletions seen in the analyzed cloning strains, all encompassing the *lac* operon and tracing back to the *E. coli* strains developed and investigated in the laboratory of F. Jacob [28], the mechanism of formation of the F′‐plasmid proposed here for XL1‐Blue (see Fig. 3) could provide a common explanation. In this two‐step process, first, the native F‐plasmid integrates into the chromosome of wild‐type *E. coli* in the vicinity of the *lac* region, thus forming an Hfr strain. Second, this mobile element gets excised, as F′‐plasmid, taking with it large flanking chromosomal regions on both sides, including the *lac* operon. This excision is apparently mediated by short homologous sequence stretches within the genome (REP*17a* and REP*32b* elements in case of the XL1‐Blue F′‐plasmid). This genetic recombination leaves behind a modified *E. coli* strain carrying the corresponding chromosomal deletion. This mechanism conforms with the generation of so‐called F‐Lac^+^ episomes during the construction of *E. coli* Hfr strains [28]. In fact, a closer look into the genomic DNA sequences of the strains discussed here as well as some related cloning strains, in particular DH5α [78] and DH10B [26], and their comparison with the MG1655 reference strain reveals always short repeated sequence stretches around the junctions of the chromosomal deletions (Table 3). This is consistent with the notion that the ancestor strains X111, X74, U169 [28] (and also SL10 [2]) were all derived via distinct excision events from one (or a few) Hfr progenitor(s) carrying the F‐plasmid inserted close to the *lac* locus, initially generated at the Pasteur Institute (Paris, France).

**Table 3:** Large chromosomal deletions in *E. coli* cloning strains encompassing the *lac* region.

Cloning strain	Ancestor	Deleted region	Nucleotide range^a^	Recombination site
JM83	X111	*gpt*–*mhpC*	256 104–371 377	CCGG↓GTGC
JM109	SL10	*lfhA*–*mhpE*	249 546–373 869	GCGGCAT↓
DH5α	U169	*mmuP*–*mhpD*	275 509–372 748	GTCTGGCTGG↓
DH10B	X74	*yahG*–*mhpE*	339 199–375 417	TGGC(G/A)ATGT↓

^a^ in the *E. coli* K‐12 reference genome, MG1655.

Hence, the present genome sequence analysis of commonly used laboratory strains of *E. coli* offers plausible mechanisms for many of the manipulations from classical bacterial genetics [21] that were applied during their construction. On the other hand, it provides evidence of the remarkable genetic stability of the severe chromosomal and episomal alterations despite their introduction with the help of mobile genetic elements whose remnants are still detectable. However, a number of so far mostly unknown accompanying changes have also become apparent. Of course, the toolbox of modern molecular biology offers more precise means of chromosomal DNA manipulation to achieve similar goals as with these classical laboratory strains. In this regard, the genome sequence analysis of JM83, JM109, and XL1‐Blue should provide useful hints for the construction of improved *E. coli* strains in the future. Beyond such practical considerations, it deserves tribute how efficiently many useful traits were combined in these widely employed *E. coli* derivatives by the pioneers of bacterial genetics.

## Conflict of interest

The authors declare no conflict of interest.

### Peer review

The peer review history for this article is available at https://www.webofscience.com/api/gateway/wos/peer‐review/10.1002/2211‐5463.13812.

## Author contributions

AS conceived and supervised the study. SA performed experiments. AS and SA analyzed data. AS wrote the manuscript. SA and AS edited and approved the final manuscript version.

## Supporting information


**Table S1.** Previously unknown genotypic features of JM83.

**Table S2.** Previously unknown genotypic features of XL1‐Blue.

**Table S3.** Prominent genotypic differences between JM109 and XL1‐Blue.

**Data S1.** Annotated JM83 chromosome DNA sequence file (generated with snapgene version 7.1.2 software).

**Data S2.** Annotated XL1‐Blue chromosome DNA sequence file (generated with snapgene version 7.1.2 software).

**Data S3.** Annotated XL1‐Blue episome DNA sequence file (generated with snapgene version 7.1.2 software).

## Data Availability

The data that support the findings of this study are openly available from NCBI GenBank at https://www.ncbi.nlm.nih.gov, reference number PRJNA740136, and as supplementary material of this article (Supporting Information).
