# Supplementary material for: Comparative genome analysis of three classical E. coli cloning strains designed for blue/white selection: JM83, JM109 and XL1‐Blue
Source: FEBS Open Bio. 2024 May 10;14(6):888–905. doi: 10.1002/2211-5463.13812 (PMC11148124; doi:10.1002/2211-5463.13812)
Supplement: Supplementary file 1 — Table S1. Previously unknown genotypic features of JM83. [file FEB4-14-888-s001.pdf]

Table S1: Previously unknown genotypic features of JM83

Gene name	Changes in JM83 *versus E. coli* K-12 MG1655	Gene product
icd$^+$	no insertion of the cryptic prophage e14	isocitrate dehydrogenase (intact gene product)
opgD	frameshift (Δ1 bp)	glucan biosynthesis protein D
rfbD	frameshift (Δ1 bp)	dTDP-4-dehydrorhamnose reductase
gatC$^+$	frameshift (Δ2 bp) leads to an intact reading frame and a full-length 451 aa gene product	galactitol-specific PTS enzyme IIC component (inactive in MG1655 due to stop codon at position 312)
gatY	insertion of IS*4*	tagatose-1,6-bisphosphate aldolase 2 SU GatY
yeiS	nonsense mutation: stop codon at aa 4	DUF2542 domain-containing protein YeiS
mglA	insertion of IS*186*	D-galactose/methyl-galactoside ABC transporter ATP binding subunit
lrhA	frameshift (+1 bp)	DNA-binding transcriptional dual regulator LrhA
evgS	frameshift (Δ1 bp)	sensor histidine kinase EvgS
xanQ	frameshift (Δ1 bp)	xanthine:H^+ symporter XanQ
ttdB	frameshift (Δ1 bp)	L(+)-tartrate dehydratase subunit β
yhdJ	nonsense mutation: stop codon at aa 13	DNA adenine methyltransferase
glpR$^+$	frameshift (+1 bp) leads to an intact reading frame and a full-length 252 aa gene product	DNA-binding transcriptional repressor GlpR (inactive in MG1655 due to deletion of C directly after codon Leu50)
xylA	nonsense mutation: stop codon at aa 69	xylose isomerase
mtlA	frameshift (Δ1 bp)	mannitol-specific PTS enzyme IICBA component
rph$^+$	frameshift (+1 bp) in codon Gly 223 leads to an intact reading frame and a full-length 238 aa gene product	RNase PH (reading frame C-terminally truncated by 10 aa in MG1655)
cytR	insertion of IS*2*	DNA-binding transcriptional repressor CytR
ytfI	nonsense mutation: stop codon at aa 62	protein YtfI
