# Supplementary material for: Comparative genome analysis of three classical E. coli cloning strains designed for blue/white selection: JM83, JM109 and XL1‐Blue
Source: FEBS Open Bio. 2024 May 10;14(6):888–905. doi: 10.1002/2211-5463.13812 (PMC11148124; doi:10.1002/2211-5463.13812)
Supplement: Supplementary file 2 — Table S2. Previously unknown genotypic features of XL1‐Blue. [file FEB4-14-888-s006.pdf]

Table S2: Previously unknown genotypic features of XL1-Blue

**Gene name**	**Changes in XL1-Blue *versus E. coli* K-12 MG1655**	**Gene product**
sgrR	nonsense mutation: stop codon at position 351	DNA-binding transcriptional dual regulator SgrR
guaC	nonsense mutation: stop codon at aa 84	GMP reductase
crl^{+}	no insertion of IS*1*: intact gene product	RNA polymerase holoenzyme assembly factor Crl
ykgV	insertion of IS*5*	Protein YkgV of cryptic prophage CP4-6
DLP12	deletion from within *intD* up to *essD* / insertion of IS*5*	cryptic prophage DLP12
glnX	nt 35 G→A	supE44 / amber suppressor tRNA
pgaA	insertion of IS*5*	partially deacetylated poly-β-1,6-N-acetyl-D-glucosamine outer membrane porin
serX	duplication of a 181 bp sequence leads to a 2nd copy	tRNA-Ser
intE	insertion of IS*3*	cryptic prophage e14
ycfK–stfE	precise inversion of a 1,797 bp segment, flanked by a 16 nt inverted repeat (TTGGTTTGGGAGAAGG)	TfaE/P as part of the cryptic prophage e14
narG	nonsense mutation: stop codon at aa 922	nitrate reductase A subunit α
rssB	nonsense mutation: stop codon at aa 143	regulator of RpoS
IS*5U*	insertion sequence deleted	intergenic region *ychE...oppA*
tonB	insertion of IS*10*	Ton complex subunit TonB
fnr...ogt	insertion of IS*5*	intergenic region
abgB	nonsense mutation: stop codon at aa 455	p-aminobenzoyl-glutamate hydrolase subunit B
paaA	nonsense mutation: stop codon at aa 22	phenylacetyl-CoA 1,2-epoxidase, monooxygenase subunit
yncI	insertion of IS*10*	putative transposase YncI (truncated due to stop codon)
kdgR	insertion of IS*5*	DNA-binding transcriptional repressor KdgR
IS*1H*	insertion sequence deleted	intergenic region *flhD...uspC*
rfbD	frameshift (Δ1 bp)	dTDP-4-dehydrorhamnose reductase

gatC^{+}	frameshift (Δ2 bp) leads to an intact reading frame and a full-length 451 aa gene product	galactitol-specific PTS enzyme IIC component (inactive in MG1655 due to stop codon at position 312)
gatB	insertion of IS*5*	galactitol-specific PTS enzyme IIB component
REP*161c/d*	deletion of 113 bp	intergenic region
eutA...eutB	no insertion of cryptic prophage CPZ-55	ethanolamine ammonia-lyase EutBC / reactivating factor
rrlG	4 nt substitutions	23S ribosomal RNA
luxS	frameshift (Δ1 bp)	S-ribosylhomocysteine lyase
rpoS	nonsense mutation: stop codon at aa 270	RNA polymerase, sigma S (sigma 38) factor
ygeY	nonsense mutation: stop codon at aa 268	putative peptidase YgeY
ftsP	nonsense mutation: stop codon at aa 152	cell division protein required during stress conditions
ttdB	frameshift (Δ1 bp)	L(+)-tartrate dehydratase subunit β
glpR^{+}	frameshift (+1 bp) leads to an intact reading frame and a full-length 252 aa gene product	DNA-binding transcriptional repressor GlpR (inactive in MG1655 due to deletion of C directly after codon Leu50)
IS*1E*	nonsense mutation: stop codon at aa 98	IS*1* protein InsB
IS*5T*	nonsense mutation: stop codon at aa 140	IS*5* transposase and trans-activator InsH
rph^{+}	frameshift (+1 bp) in codon Gly 223 leads to an intact reading frame and a full-length 238 aa gene product	RNase PH (reading frame C-terminally truncated by 10 aa in MG1655)
bglH	nonsense mutation: stop codon at aa 537 (results in loss of a C-terminal double-Trp tag)	carbohydrate-specific outer membrane porin, cryptic
rbsR	frameshift (Δ1 bp)	DNA-binding transcriptional dual regulator RbsR
REP*299f*	34 bp Insertion	
thrT	mutation of 3'-end CCA→TCA (non-functional tRNA)	tRNA-Thr
REP*321i/j*	deletion of 111 bp	
cadB	nonsense mutation: stop codon at aa 41	lysine:cadaverine antiporter
cpdB	insertion of IS1 and stop codon at aa 481	2',3'-cyclic nucleotide phosphodiesterase/3'-nucleotidase
fimE	insertion of IS1	regulator for fimA
