# Supplementary material for: Comparative genome analysis of three classical E. coli cloning strains designed for blue/white selection: JM83, JM109 and XL1‐Blue
Source: FEBS Open Bio. 2024 May 10;14(6):888–905. doi: 10.1002/2211-5463.13812 (PMC11148124; doi:10.1002/2211-5463.13812)
Supplement: Supplementary file 3 — Table S3. Prominent genotypic differences between JM109 and XL1‐Blue. [file FEB4-14-888-s005.pdf]

Table S3: Prominent genotypic differences between JM109 and XL1-Blue

Gene name	Changes in JM109 *versus* XL1-Blue	Gene product
sokC	insertion of IS*150*	small regulatory RNA antitoxin SokC
lfhA–mhpF	124,324 bp deletion (nt 249,546–373,869 in MG1655)	Δ(*lac-pro*) – differs from the deletions in X74 and X111
clpX...lon	insertion of IS*186*	intergenic region
$pgaA^{+}$	no insertion of IS*5*	partially deacetylated poly-β-1,6-N-acetyl-D-glucosamine outer membrane porin
icd^{+}	no insertion of the cryptic prophage e14	isocitrate dehydrogenase
$tonB^{+}$	no insertion of IS*10*	Ton complex subunit TonB
pspF	N-terminal deletion of 176 bp up to codon Arg55	DNA-binding transcriptional dual regulator PspF
$yncI^{+}$	no insertion of IS*10*	putative transposase YncI (truncated due to stop codon)
ydgI	insertion of 33 bp after codon Glu2	putative arginine:ornithine antiporter
$kdgR^{+}$	no insertion of IS*5*	DNA-binding transcriptional repressor KdgR
menD	insertion of IS*5*	2-succinyl-5-enolpyruvyl-6-hydroxy-3-cyclohexene-1-carboxylate synthase
$luxS^{+}$	no frameshift	S-ribosylhomocysteine lyase
recA	missense mutation L78P (CTG→CCG) instead of the mutation G161D	DNA recombination/repair protein RecA
srlD	insertion of both IS*10L* and IS*10R* directly in tandem	sorbitol-6-phosphate 2-dehydrogenase
$rpoS^{+}$	no stop codon	RNA polymerase, sigma S (sigma 38) factor
ygeH	insertion of IS*5*	putative transcriptional regulator YgeH
$ygeY^{+}$	no stop codon	putative peptidase YgeY
yhcG	insertion of IS*2*	DUF1016 domain-containing protein YhcG
yicL	insertion of IS*10*	putative inner membrane protein
